# Possible Founder Effect of the CDKN2A c.146T>C Variant in the Mexican Population: Phenotypic Characterization

**DOI:** 10.3390/genes17050532

**Published:** 2026-04-30

**Authors:** María Lourdes Garza-Rodríguez, Eren Adrián Alejandro Vargas-Márquez, Dione Aguilar, Samantha Trujillo-Bornios, Hugo Leonid Gallardo-Blanco, Oscar Vidal-Gutiérrez, Diana Cristina Pérez-Ibave, Carlos Horacio Burciaga-Flores

**Affiliations:** 1 Servicio de Oncología, Centro Universitario Contra el Cáncer (CUCC), Hospital Universitario “Dr. José Eleuterio González”, Universidad Autónoma de Nuevo León, Monterrey 66451, Nuevo Leon, Mexico; maria.garzarg@unal.edu.mx (M.L.G.-R.); evargasm2400@alumno.ipn.mx (E.A.A.V.-M.); stbbts13@gmail.com (S.T.-B.); hugo.gallardobl@uanl.edu.mx (H.L.G.-B.); oscar.vidalgtz@uanl.edu.mx (O.V.-G.); 2 Laboratorio de Medicina de Conservación, Centro de Biotecnología Genómica, Instituto Politécnico Nacional (IPN), Reynosa 88710, Tamaulipas, Mexico; 3 Breast Cancer Center, Hospital Zambrano Hellion TecSalud, San Pedro Garza Garcia 66260, Nuevo Leon, Mexico; dra.dioneaguilar@tecsalud.mx; 4 Instituto Mexicano del Seguro Social (IMSS), Hospital de Ginecología y Obstetricia No. 23, Monterrey 64000, Nuevo Leon, Mexico

**Keywords:** *CDKN2A*, germline variant, founder effect, breast cancer, hereditary cancer

## Abstract

**Background:** Germline *CDKN2A* variants are associated with Familial Atypical Mole-Malignant Melanoma (FAMMM) syndrome. This syndrome involves an increased risk of melanoma, pancreatic cancer, and, in specific populations, duodenal cancer, breast cancer, and astrocytoma. The *CDKN2A* (c.146T>C) variant has been found in hereditary cancer patients within the Mexican population. Furthermore, the phenotype linked to this variant in Mexico differs from that observed in other groups. This study aims to evaluate the founder effect of the *CDKN2A* (c.146T>C) variant through epidemiological analysis and to describe the phenotype within our population. **Patients and Methods**: We examined 72 Mexican patients (14 probands from distinct families, 48 relatives, and 10 nonrelated probands) carrying the *CDKN2A* (c.146T>C) form three hereditary cancer centers between September 2023 and September 2025. **Results:** Of the 72 individuals analyzed, 52 (72.22%) tested positive. A cancer diagnosis was established in 27 (37.50%) of the individuals analyzed. Breast cancer was the most common neoplasia, accounting for 19 cases (70.37%), followed by melanoma with 4 cases (14.81%) and ovarian cancer with 2 cases (7.40%). Three patients (11.11%) had two distinct primary neoplasms. **Conclusions:** Based on our findings and the fact that this variant has been reported nearly exclusively in the Mexican population, we conclude that it has a founder effect in this population. Additionally, the phenotype associated with this variant can vary among populations, with breast cancer being the most common carcinoma rather than melanoma among Mexican carriers, highlighting the importance of updating screening guidelines.

## 1. Introduction

Familial Atypical Multiple-Mole Melanoma (FAMMM) is an autosomal dominant hereditary cancer syndrome marked by familial melanoma and atypical nevi [1], caused by germline variants in *CDKN2A*. Besides melanoma, *CDKN2A* carriers also have higher risks of other cancers, like pancreatic cancer [2].

The *CDKN2A* gene spans approximately 27.5 kilobases (kb) and is located on chromosome 9p21. This gene encodes two different tumor suppressor proteins (p16INK4A and p14ARF) via alternative splicing. It comprises four exons (1α, 1β, 2, and 3). Exons 1α, 2, and 3 contribute to the production of the p16 protein, which binds to cyclin-dependent kinase 4 (CDK4) and prevents its interaction with cyclin D. This interaction prevents phosphorylation of the retinoblastoma protein and, as a result, halts the transition from the G1 to the S phase in the restriction point of the cell cycle. Exons 1β, 2, and 3 encode the p14ARF (p14) protein, with the main role of stabilizing p53 by inhibiting its MDM2-induced degradation, preserving the heterodimer [1,2,3]. Germline *CDKN2A* variants seen in melanoma and pancreatic cancer-prone families are typically missense or nonsense mutations that impair the inhibitory function of the p16 protein [4], thereby disrupting cell-cycle regulation and leading to uncontrolled cell growth and proliferation. Pathogenic variants in this gene have been linked with an increased risk of various neoplasms, primarily to an increased risk of melanoma in 22–40% and pancreatic cancer in 17%. However, there are some reports of other related neoplasms, including lung, head and neck, gastrointestinal, breast, sarcomas, and multiple myeloma [2,3,5].

The frequency of germline *CDKN2A* pathogenic variants varies across populations, ranging from approximately 0.6% to 7.8%, depending on the population analyzed, as observed in patients with pancreatic cancer or melanoma [6,7,8].

In the Latino population, Balderas et al. found that the frequency of pathogenic *CDKN2A* variants associated with melanoma ranges from 8.2% to 9%. This report includes some recurring variants, such as the c.142C>A (p.P48T) variant found in European, Brazilian, and Italian populations.

In Chilean patients, the variant c.430C>T (p.R144C) has been linked to a possible founder effect, as it has been found mainly in this population. Interestingly, this report also showed that another variant, c.146T>C (p.I49T), was found in two Mexican patients and was not observed in other subjects of any of the studied populations [9]. The p.I49T variant is in the coding exon 1 of the *CDKN2A* gene, resulting from a T-to-C substitution at nucleotide 146.

In another study conducted by Puig et al., who analyzed a large cohort of 186 Latin American patients, three Mexican patients with familial melanoma were identified. Of these, two (66.6%) carried the c.146T>C (p.I49T) variant. It is also worth noting that this variant was not found in any of the other ethnic groups [9]. Finally, a study by Kathryn A. Mraz, involving a sample of 123 patients with the c.146T>C (p.I49T) variant, found that 80% had Latino ancestry in at least one family line, though their countries of origin were not specified, suggesting a founder effect. This report also describes an atypical phenotype according to the established cancer risk for the gene, including a person with breast cancer at age 30, pancreatic adenocarcinoma at age 50, and a family history of male breast cancer and brain tumor [10]. Historically, in ClinVar, the *CDKN2A* c.146T>C (p.I49T) variant was reported as a variant of uncertain significance (VUS); most recently, on 31 January 2026, the classification was changed to likely pathogenic [11]. In addition, there is strong evidence that this variant is responsible for several neoplasms in carriers [9,11,12]. As previously shown, the *CDKN2A* c.146T>C (p.I49T) variant has been identified primarily in patients of Mexican ancestry with pancreatic cancer or melanoma, and, to our knowledge, no other studies have reported this variant in other populations [9].

Lauren Gima and colleagues examined the c.146T>C (p.I49T) variant in the global population. They found that the variant is more common among the Hispanic population than other groups, with most carriers having at least one parent of Mexican descent (94%), supporting the founder effect hypothesis. In this study, they only identified the classic FAMMM syndrome phenotype in this population, as their reported neoplasms included pancreatic cancer and melanoma [13]. There are a few reports from Mexican populations; one includes a 14-year-old with osteosarcoma who carried the variant in a homozygous state, along with a *MUTYH* variant [14]. Additionally, there is an abstract by Daisy Hernández and colleagues, presented at AACR (American Association for Cancer Research), which discusses this variant; they identified the c.146T>C (p.I49T) variant in 92 Mexican patients with a personal or family history of pancreatic cancer or melanoma, further supporting the founder effect of the variant [12], but not expanding the phenotype spectrum of the variant.

Studies on the Mestizo Mexican population are important for understanding the distribution of cancer risk variants. Detecting germline variants in the Mexican population plays a crucial role in cancer prevention and treatment. Mexico, as a developing country, can benefit from genetic counseling and genomic testing for pathogenic variants, helping unlock the potential of personalized medicine for its people.

## 2. Materials and Methods

### 2.1. Sample Population and Approval from the Ethics Committee

This study was carried out at the Centro Universitario Contra el Cáncer (CUCC) of the Hospital Universitario “Dr. José Eleuterio González” of the Universidad Autónoma de Nuevo León, following the Declaration of Helsinki. The protocol was approved by the Institutional Ethics Committee of the University Hospital “Dr. José Eleuterio González” under registration number ON18-00015.

All participants signed an informed consent form after agreeing to participate in the study. Patients were recruited from the Instituto Mexicano del Seguro Social (IMSS) Hospital de Ginecología y Obstetricia #23 (HGO # 23), Hospital Universitario “Dr. José Eleuterio González”(HU), and Tec Salud Breast Cancer Center, the only private institution. Patients from HGO 23 were referred from multiple oncology services of the northeastern IMSS grid. HU public health patients were referred from the University Cancer Center. Tec Salud Breast Cancer Center patients received private patients from the Nuevo León metropolitan area. All included institutions provide oncology and genetic services for all neoplasms and possible hereditary cancer syndromes. Subsequently, clinical and epidemiological information from patients was collected, and blood samples were obtained. A total of 72 individuals (14 probands from distinct families, 48 relatives, and 10 nonrelated probands) referred to our center for genetic counseling between September 2023 and September 2025 were assessed (Table S1). Clinical data from non-analyzed relatives were collected to describe the nonspecific phenotype (Table S2). Patients were referred to medical genetics consultation from the different oncology services of the participant institutions according to the NCCN guidelines for high-risk individuals, mainly based on age of diagnosis, tumor type, and family history of cancer. Hereditary cancer diagnosis was established according to the American College of Medical Genetics (ACMG) [15] and the National Comprehensive Cancer Network (NCCN) guidelines [16].

### 2.2. Genetic Counseling

Before protocol inclusion, patients and relatives suspected of hereditary cancer syndrome had genetic counseling. All probands underwent genetic testing, including an 84-gene Invitae Multi-Cancer Panel (San Francisco, CA, USA); a 30-gene Onco Life test^®^ from Life in Genomics^®^ (Mexico City, Mexico); whole-exome sequencing with Illumina (San Diego, CA, USA); and multi-panel testing with the 113-gene TruSight Hereditary Cancer Panel (Illumina, San Diego, CA, USA). All panels included *CDKN2A* exome gene sequencing. After the test results arrived, post-test genetic counseling was offered for disclosure of positive or negative testing. Positive patients received detailed, personalized explanations about the affected gene, treatment options, screening methods, risk-reduction strategies, and cancer prevention. If the test was negative, the patient was discharged and continued their oncologic treatment or follow-up, as appropriate. This patient group is part of a larger cohort that includes individuals with various hereditary cancer syndromes [17,18,19]. For patients carrying the *CDKN2A* c.146T>C variant, the option to participate in the study and to have their relatives participate was offered. Those who accepted signed the informed consent and were enrolled in the protocol, received genetic counseling, and had their relatives offered Sanger sequencing for all variants found in the probands, including those with two or more variants.

### 2.3. DNA Extraction

Peripheral blood samples (5 mL) were collected via venipuncture, and no adverse events from the puncture were reported. Genomic DNA was extracted from leukocytes using the QIAamp DNA Blood Midi kit (QIAGEN, Hilden, Germany), following the manufacturer’s instructions. The DNA concentration was measured by optical density (OD) at 260 nm with the QIAxpert UV/Vis spectrophotometer (QIAGEN, Hilden, Germany). The OD260/OD280 ratio was used to evaluate the DNA purity, with values between 1.8 and 2 indicating high purity. Genomic DNA was stored at −80 °C until required.

### 2.4. Primer Design and PCR Amplification

The sequence of interest rs199907548 was retrieved from the public database NCBI [11]. The flanking sequence includes at least 500 nucleotides, both upstream and downstream of the variant, and excludes other polymorphic variants to prevent primer design in variable regions. Specific primers for the *CDKN2A* germline variant (p16INK4a), c.146T>C (p.I49T), were designed using AmplifX 2.1.1 software [20], which was also used for amplification simulation. Primer specificity was confirmed with Primer-BLAST (Primer 3 v.2.5.0 from NCBI) [21].

Amplification reactions were carried out using the GoTaq Colorless PCR Master Mix (Promega, Madison, Wisconsin, USA), with 10 μM of each primer (Forward 5′- CCAGGTATCTGTGAATTGGAGGCTAAGTAGTC -3′ and Reverse 5′- TTCCGCCAGCACCGGAGGAAGAAAG -3′), along with 100 ng of genomic DNA from each sample, in a final reaction volume of 50 μL. PCRs were performed in a SimpliAmp Thermal Cycler (Applied Biosystems, Foster City, CA, USA). The amplification program included an initial denaturation step of 2 min at 95 °C, followed by 40 cycles of 45 s each at 95 °C, 45 s at 65 °C, and 45 s at 72 °C, ending with a final elongation step of 5 min at 72 °C. The 654 bp amplification products were visualized on 1% agarose gels stained with SYBR Safe DNA Gel Stain (Invitrogen, Thermo Fisher Scientific, Waltham, MA, USA) and observed under UV light.

### 2.5. Sanger Sequencing

The PCR products were purified using the Wizard^®^ SV Gel and PCR Clean-Up System (Promega, Fitchburg, WI, USA) following the manufacturer’s instructions. Next, the PCR products were sequenced using BigDye Terminator v1.1 cycle sequencing reagents, and the products were purified using a BigDye XTerminator purification kit (Thermo Fisher Scientific, Waltham, MA, USA) according to the manufacturer’s guidelines. The sequencing reactions were analyzed on a SeqStudio Genetic Analyzer (Thermo Fisher Scientific, Waltham, MA, USA). For sequence analysis, we used Sequencing Analysis v7 and SeqScape v4 software (Applied Biosystems, Foster City, CA, USA).

### 2.6. Statistical Analysis

Statistical analysis in Microsoft Excel v.10 was performed, including descriptive statistical measures such as mean, standard deviation (SD), mode, and median. Student’s *t*-test for continuous variables and Fisher’s exact test for categorical variables were used to assess significance. All analyses were two-tailed, with *p* < 0.05 considered significant.

## 3. Results

**Figure 1:**
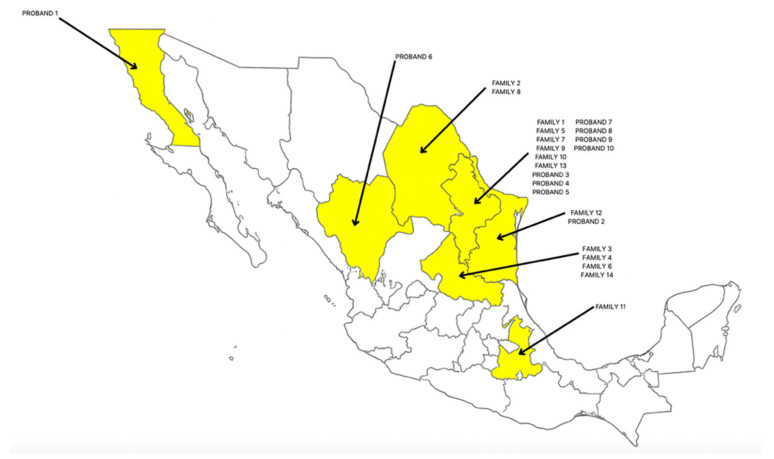
Distribution of families and unrelated individuals studied in the different states of Mexico. There was only one family from the center of the country (the state of Puebla); the rest came from northern states.

In this study, we examined 72 individuals for the *CDKN2A* c.146T>C (p.I49T) variant. We analyzed a cohort that included 14 families and 10 unrelated individuals with personal or family history of cancer. In the analysis of demographic data for participants, we describe the sex distribution: 56 (77.77%) women and 16 (22.22%) men. The average age for molecular analysis was 47.8 years for the probands and 42.8 years for their relatives. All participants were Mexican, primarily from northern Mexico (69), including Nuevo León, Coahuila, Durango, San Luis Potosí, Tamaulipas, and Baja California Norte. There was only one family from the central region (Puebla) (Figure 1).

A cancer diagnosis was established in 27 (37.50%) of the individuals analyzed, including 24 (88.88%) of the probands and 3 (11.11%) of the relatives. The average age at diagnosis was 43.68 years, with a range from 25 to 74 (Table 1). Among the reported neoplasms, we found that breast cancer was the most common, accounting for 19 cases (70.37%), followed by melanoma with 4 cases (14.81%) and ovarian cancer with 2 cases (7.40%). There were three patients (11.11%) who had two distinct primary neoplasms at the time of diagnosis: one of them had two melanomas, with the first diagnosed at 23 years and the second at 27 years old. Another patient had a synchronic tumor diagnosis with NTNBC and melanoma at 55 years old, and the last one had hepatic and kidney carcinoma at 37 and 38 years of age. Also, one of the relatives had two premalignant skin lesions removed (dysplastic nevi) (Table 2)

Cancer staging within our cohort was mainly at early or intermediate levels. None of our patients presented with metastatic cancer at diagnosis. Analyzing specifically, for breast cancer, as the most reported neoplasia, we found that there were 11 (57.89%) cases diagnosed at an early stage (clinical stage I and II), and 5 (26.32%) at an intermediate stage (clinical stage III) (Table 1). Regarding the molecular subtype of breast cancer, we found that luminal type breast cancer was the most diagnosed, representing 13 (68.42%) of the cases, while the remaining cases were triple-negative breast cancer, totaling 6 (31.57%).

Patients reported family history of cancer in 20 cases: pancreatic cancer was the most common in 4 (20%) cases, followed by gastric cancer in 3 (15%) cases, other important reported neoplasms included breast carcinoma and melanoma in 2 (10%) cases each. There was also one relative that had three primary tumors (melanoma, liposarcoma, and breast carcinoma) (Table 2).

The *CDKN2A* c.146T>C (p.I49T) variant was found in 52 (72.22%) of the individuals, while 20 (27.78%) tested negative for the variant or for any other related gene. Interestingly, we identified five patients carrying two pathogenic variants, a condition known as MINAS (Multi-locus Inherited Neoplasia Allele Syndrome): one individual is homozygous for the *CDKN2A* c.146T>C (p.I49T) variant; another patient and her brother carries both *BRCA1* c.5123C>A (p.Ala1708Glu) and *CDKN2A* c.146T>C (p.I49T) variants; another patient has *BRCA1* del ex 9–12 along with *CDKN2A* c.146T>C (p.I49T) variants; and one patient has *PALB2* c.2411_2412del in combination with *CDKN2A* c.146T>C (p.I49T) (Table S3).

## 4. Discussion

Some previous studies have indicated that the *CDKN2A* c.146T>C (p.I49T) variant likely has a founder effect in the Mexican population. In this study, we sought to strengthen the role of this variant as a possible founder in the Mexican population. Based on available epidemiological and clinical evidence of our Mexican patient cohort, in which we found a distinct phenotype that includes breast cancer as a common tumor, exceeding pancreatic cancer and melanoma in frequency. We propose that this variant is involved in the pathogenesis of a wide spectrum of tumors within the syndrome. To corroborate this, a larger cohort is required. The range of mutations affecting *CDKN2A* is crucial for understanding their clinical importance [3]. To date, 1656 germline variants have been documented in the ClinVar database, of which 214 have been classified as pathogenic [11]. The p.I49T variant (also known as c.146T>C), located in coding exon 1 of the *CDKN2A* gene, results from a T to C change at nucleotide 146. The isoleucine at codon 49 is replaced by threonine, an amino acid with similar properties [11]. Research into the functional effects of variants in this region shows that they decrease p16’s ability to bind to CDK4 and CDK6 and impair its capacity to influence Rb dephosphorylation, leading to uncontrolled cell proliferation [22,23]. Despite prediction tools indicating that the variant is deleterious, it is reported in ClinVar as having uncertain significance due to a lack of demonstrated clinical relevance in some populations. However, in our population, it shows a pathogenic effect, underscoring the need to continue exploring personalized treatment approaches for individuals with *CDKN2A* mutations.

The PAGE (Population Architecture using Genomics and Epidemiology) study, in ClinVar, which further categorized the Latin population, reports that this variant was mainly present in the Mexican population (n = 10808, A = 0.99565; G = 0.00435) and also in the Native American population (n = 1260, A = 0.9992; G = 0.0008) [10,12]. In a previous report by our group, we identified that the most common non-*BRCA* genes linked to hereditary breast cancer include *CHEK2*, *PALB2*, *MUTYH*, *CDKN2A*, and *ATM*. In our cohort, we identified 10 cases involving *CDKN2A* variants; among them, 6 carried the c.146T>C (p.I49T) variant, providing strong evidence of its high frequency in Mexican patients [18]. We found five patients with two pathogenic variants, including the *CDKN2A* variant studied, who exhibited no differences in clinical presentation, age, or treatment response, consistent with previous studies (Table S3) [19].

Of the five MINAS cases, three patients had a breast cancer diagnosis, and one had melanoma (Table S3). The patient with melanoma is a homozygous patient for the *CDKN2A* variant; she had developed two melanomas at the time of diagnosis. In autosomal dominant diseases, having pathogenic variants in a homozygous state is expected to have a more severe phenotype; however, as we have only one patient, we cannot determine the reach for the genotype. The remaining three patients with breast carcinoma had pathogenic variants in the *BRCA1* and *PALB2* genes. Both genes are known to have a stronger relationship with an increased breast carcinoma risk, especially with the triple-negative molecular subtype. Analyzing our breast cancer cohort, we found that the molecular subtype of the MINAS cases corresponds to triple-negative ones, which is concordant with the classical association of *BRCA1* and *PALB2* with breast cancer; meanwhile, in 16 cases (84.21%) with the *CDKN2A* variant, only 3 cases (18.75%) had a triple-negative phenotype. Due to the low frequency of the gene and the unusual genotype of MINAS patients, establishing a clear phenotype can be difficult, so a larger cohort is needed. Genetic counseling for MINAS patients and their relatives was based on the current evidence, which assumed that having two pathogenic variants had an additive rather than a synergistic effect [19], taking the risk for tumors for the more penetrant gene and the cumulative risk for tumors for the screening.

In this study, we examined 14 families and 10 unrelated individuals with the variant. We suggest a possible founder effect, likely originating in the north, given that most patients were from this region. However, due to the limited sample size, further studies in other Mexican cohorts are needed to confirm the variant’s origin, as our sample may be biased by the study centers’ locations.

*CDKN2A* has traditionally been linked to pancreatic cancer and melanoma as part of the FAMMM syndrome [13]. The current nomenclature for the FAMMM syndrome does not cover the whole spectrum of neoplasms linked to *CDKN2A* variants, such as non-melanoma skin cancer, non-pancreatic gastrointestinal cancer, breast cancer, and other less frequent tumors like nervous system or lung cancers. Previous evidence has associated *CDKN2A* variants with different cancers than melanoma, including non-pancreatic gastrointestinal, skin, lung, and breast cancers, with an increased risk for breast cancer of 1.9–3.8 [2,3,24].

We observed an unusual presentation in our cohort with the c.146T>C (p.I49T) variant, with breast carcinoma reported as the common neoplasm, representing 19 (70.37%) cases, which is higher than the classical reported neoplasia in the FAMMM syndrome. In our cohort, we also found four (14.81%) cases of melanoma, two (7.40%) cases of ovarian cancer, and a single case (3.70%) of pancreatic cancer. Additionally, there were less common neoplasms like ovarian, endometrial, liver, and kidney cancers. The result of this study supports the worldwide evidence that *CDKN2A* increases risk for a broader spectrum of neoplasms and limiting it to the pancreas and melanoma could overlook several diagnoses.

We suggest “*CDKN2A* cancer predisposition syndrome” as a more accurate term. Using ‘syndrome’ may help clinicians recognize risks beyond melanoma and pancreatic cancer.

In our study, all participant centers include patients with any type of neoplasm; however, breast cancer is the main reason for oncology consultations in our country [25]. Also, the most frequently associated cancer with hereditary cancer syndromes is by an oncologist, which may explain a possible bias in our cohort’s patients.

Breast cancer is one of the most common carcinomas, with a worldwide incidence of 2,296,840 cases and 666,103 deaths reported in 2022, according to GLOBOCAN. In Mexico, breast cancer remains the most common neoplasia, with 31,043 annual cases representing up to 15% of all neoplasms for both sexes [25]. Up to 10–15% of breast carcinomas are estimated to be part of a hereditary cancer syndrome. In a previous study, we reported that among the non-*BRCA* genes involved in hereditary breast cancer, *CDKN2A* ranked fourth in frequency of involvement [18]. Regarding breast molecular subtypes, we found that 13 (68.42%) of our cases were luminal subtype, which differs from the classic association seen in *BRCA*-positive breast cancer, where triple-negative breast cancer is most common. The association of *CDKN2A* and breast cancer is not new; multiple publications address this correlation [24,26,27,28]. Unfortunately, current international guidelines (NCCN and ESMO) do not include breast cancer screening or screening for other neoplasms for *CDKN2A* carriers, aside from pancreas and melanoma, highlighting a gap in current recommendations based on the most current and updated population-based information. Given the high frequency of this variant in our population, it is essential to implement effective risk management strategies for these patients. Analyzing the age of onset, we found that the average was 47.9 years, which is typical for a cancer predisposition gene carrier. However, the ages of onset varied widely (25–74 years), indicating moderate-to-high penetrance of the variant. Our range was so wide that one patient developed cancer at 74 years, but most patients were in their fourth decade of life. In clinical staging, we did not observe an association with a more aggressive phenotype, as none of our patients presented at an advanced stage and all responded well to treatment. Still, this may be influenced by multiple factors, including the fact that most analyzed families had a history of cancer and, therefore, were more conscious about cancer prevention and early detection.

All diagnosed patients, including previvors, were enrolled in a screening program that includes an annual evaluation by a dermatologist to detect anomalous nevi using digital dermatoscopy, an annual MRI for pancreatic cancer, mammography and ultrasound for breast cancer, and specific screening tailored to family cancer history. At the time of the study, none of the patients had developed new neoplasms, but one of the relatives had two early detected and removed nevi.

Additionally, clinical data from the other 28 relatives were collected; the variant was not analyzed mainly because of death in 20 (71.42%) individuals. We found that a wide spectrum of neoplasia is described in Table S2. Unfortunately, as we could not demonstrate the presence of the variant, they were excluded from the study.

## 5. Conclusions

In addition to this study, there is strong evidence in the global population that suggests that *CDKN2A* variants can increase the risk for a wide spectrum of cancers, not only melanoma or pancreatic carcinoma. In particular, the recurrent *CDKN2A* c.146T>C (p.I49T) variant in Mexican patients may be a population-specific risk allele for breast cancer carcinoma based on our epidemiological data. However, validation in larger cohorts of breast cancer patients with *CDKN2A* variants is necessary. Based on this information, we proposed four recommendations. First of all, to recognize the variant as a true pathogenic variant to avoid conflicts of interpretation; second, to always include *CDKN2A* in breast cancer genetic testing as in not currently widely incorporated in guidelines; third, to modify the screening guidelines for *CDKN2A* carriers to encompass breast cancer screening, as we found this neoplasia to be as prevalent as other classic neoplasms; and, finally, to avoid confusion, to rename the syndrome from hereditary pancreas–melanoma syndrome or FAMMM to *CDKN2A* cancer predisposition syndrome.

## Figures and Tables

**Table 1:** Demographic characteristics of the population, divided into probands and their relatives.

Characteristics	Total n = 72 (100%)	Probands n = 24 (33.33%)	Relatives n = 48 (66.66%)	*p* Value ^1^
Age, Mean (SD)	44.97 y (16.69)	47.87 y (14.62)	43.52 y (17.7)	0.084
Sex, n (%)				<0.001
Female	56 (77.77%)	23 (95.83%)	33 (68.75%)	
Male	16(22.22%)	1 (4.16%)	15 (31.25%)	
Cancer	27 (37.50%)	24 (100%)	3 (6.25%)	<0.001
Clinical Stage (AJCC) ^2^ n (%)				
I	8 (36.36%)	8 (36.36%)	0 (0%)	
II	8 (36.36%)	8 (36.36%)	0 (0%)	
III	6 (27.27%)	6 (27.27%)	0 (0%)	
IV	0 (0%)	0 (0%)	0 (0%)	

^1^* p* value was calculated by the t-Student test for continuous variants and Fisher’s exact test for categorical variants. ^2^ Percentages were calculated based on 22 patients with reported clinical stage; information for 7 patients was not available.

**Table 2:** Cancer frequency in probands and relatives.

Primary Tumor	Patients n = 27 (100%)	Probands n = 24 (88.88%)	Relatives n = 3 (11.11%)
Breast	19 (70.37%)	18 (75%)	1 (33.33%)
TNBC ^1^	6 (31.57%)	6 (33.33%)	0 (0%)
NTNBC ^2^	13 (68.42%)	12 (66.66%)	1 (100%)
Melanoma	4 (14.81%)	4 (16.66%)	0 (0%)
Ovarian	2 (7.40%)	1 (4.16%)	1 (33.33%)
Pancreas	1 (3.70%)	1 (4.16%)	0 (0%)
Endometrium	1 (3.70%)	0 (0%)	1 (33.33%)
**Special cases**			
Two neoplasias	3 (11.11%) *	3 (8.33%) *	0 (0%)
Premalignant lesion ^3^	1 (100%)	0 (0%)	1 (100%)

^1^ TNBC: Triple-negative breast cancer. ^2^ NTNBC: Non-triple-negative breast cancer. ^3^ A relative had two dysplastic nevi removed. * For patients with multiple neoplasms, percentages were calculated using the total of affected individuals per group.

## Data Availability

There were no new data created in this article. For more information about our publication, please contact the corresponding authors.

## Supplementary Materials

The following supporting information can be downloaded at: https://www.mdpi.com/article/10.3390/genes17050532/s1, Table S1: Variant distribution among analyzed individuals; Table S2: Cancer frequency and non-analyzed relatives; Table S3: MINAS patients distribution.

## Abbreviations

The following abbreviations are used in this manuscript:

FAMMM	Familial Atypical Mole-Malignant Melanoma
CDK4	Cyclin-dependent kinase 4
AACR	American Association for Cancer Research
CUCC	Centro Universitario Contra el Cáncer
IMSS	Instituto Mexicano del Seguro Social
PCR	Polymerase Chain Reaction
TNBC	Triple-negative breast cancer
NTNBC	Non-triple-negative breast cancer
PAGE	Population Architecture using Genomics and Epidemiology
MINAS	Multi-locus Inherited Neoplasia Allele Syndrome
GLOBOCAN	Global Cancer Observatory
NCCN	National Comprehensive Cancer Network
ESMO	European Society For Medical Oncology
MRI	Magnetic Resonance Imaging
