# Supplementary material for: Possible Founder Effect of the CDKN2A c.146T>C Variant in the Mexican Population: Phenotypic Characterization
Source: Genes (Basel). 2026 Apr 30;17(5):532. doi: 10.3390/genes17050532 (PMC13206291; doi:10.3390/genes17050532)
Supplement: Supplementary file 1 [file genes-17-00532-s001.zip › genes-4252670-supplementary.pdf]

Supplementary tables

Table S1 Variant distribution among analyzed individuals

	Sex	Age	Cancer	Age of Dx	CDKN2A (p16INK4a) c.146T>C (p.Ile49Thr) Variant	Other Detected Variant
**Family 1**						
**Proband**	F	28	NTNBC	25	Detected	Non detected
**Father**	M	56	No		Detected	Non detected
**Mother**	F	55	No		Non detected	Non detected
**Sibling 1**	F	31	No		Non detected	Non detected
**Sibling 2**	F	35	No		Detected	Non detected
**Sibling 3**	M	38	No		Non detected	Non detected
**Family 2**						
**Proband**	F	74	Melanoma	74	Detected	Non detected
**Sibling**	F	55	No		Non detected	Non detected
**Sibling**	F	50	No		Non detected	Non detected
**Sibling**	M	46	No		Non detected	Non detected
**Daughter**	F	26	No		Detected	Non detected
**Son**	M	22	No		Non detected	Non detected
**Niece**	F	34	No		Non detected	Non detected
**Nephew**	M	26	No		Non detected	Non detected
**Niece**	F	23	No		Non detected	Non detected
**Family 3**						
**Proband**	F	30	Ovarian	28	Detected	Non detected
**Father**	M	63	No		Detected	Non detected
**Mother**	F	59	No		Non detected	Non detected
**Sibiling**	F	40	No		Detected	Non detected
**Family 4**						
**Proband**	F	43	NTNBC	38	Detected	Non detected
**Sibiling**	F	45	No		Detected	Non detected
**Sibiling**	F	47	No		Non detected	Non detected
**Family 5**						
**Proband**	F	32	TNBC	30	Detected	**BRCA1 c.5123C>A (p.Ala1708Glu)**
**Father**	M	54	No		Detected	
**Mother**	F	50	Ovarian	48	Non detected	**BRCA1 c.5123C>A (p.Ala1708Glu)**
**Sibling**	M	30	No		Detected	**BRCA1 c.5123C>A (p.Ala1708Glu)**

**Family 6**						
**Proband**	F	32	NTNBC	30	Detected	Non detected
**Mother**	F	61	No		Detected	Non detected
**Sibiling**	F	38	No		Detected	Non detected
**Family 7**						
**Proband**	F	32	Melanoma	23/27	Detected	**CDKN2A (p16INK4a) c.146T>C (p.Ile49Thr)**
**Maternal Grandfather**	M	73	No		Detected	Non detected
**Mother**	F	52	No		Detected	Non detected
**Maternal Aunt**	F	51	No		Non detected	Non detected
**Maternal Aunt**	F	48	No		Non detected	Non detected
**Sibiling**	F	33	No		Detected	Non detected
**Family 8**						
**Proband**	F	61	NTNBC	58	Detected	Non detected
**Mother**	F	96	No		Non detected	Non detected
**Daughter**	F	38	No		Detected	Non detected
**Son**	M	36	No		Detected	Non detected
**Daughter**	F	34	No		Detected	Non detected
**Family 9**						
**Proband**	F	42	NTNBC	40	Detected	Non detected
**Daughter**	F	20	No		Detected	Non detected
**Family 10**						
**Proband**	F	46	NTNBC	45	Detected	Non detected
**Father**	M	85	No		Non detected	Non detected
**Sibiling**	F	61	No		Detected	Non detected
**Sibiling**	F	52	NTNBC	37	Detected	Non detected
**Son**	M	20	No		Detected	Non detected
**Son**	M	19	No		Non detected	Non detected
**Son**	M	15	No		Non detected	Non detected
**Daughter**	F	13	No		Detected	Non detected
**Family 11**						
**Proband**	F	44	Pancreas	42	Detected	Non detected
**Mother**	F	65	No		Detected	Non detected
**Sibiling**	F	42	No		Detected	Non detected
**Family 12**						
**Proband**	F	49	NTNBC	47	Detected	Non detected
**Sibiling**	F	47	No		Detected	Non detected
**Family 13**						
**Proband**	F	55	NTNBC / Melanoma	55	Detected	Non detected
**Sibiling**	M	34	Endometrial	34	Detected	Non detected

**Family 14**						Non detected
**Proband**	F	77	TNBC	74	Detected	Non detected
**Daughter**	F	52	No		Non detected	Non detected
**Daughter**	F	51	No		Detected	Non detected
**Daughter**	F	50	No		Non detected	Non detected
**Daughter**	F	38	No		Detected	Non detected
**Non related probands**						
**Individual 1**	F	55	Melanoma	28	Detected	Non detected
**Individual 2**	F	56	NTNBC	55	Detected	Non detected
**Individual 3**	F	38	Hepatic / Kidney	37 /38	Detected	Non detected
**Individual 4**	F	64	TNBC	64	Detected	Non detected
**Individual 5**	F	36	TNBC	36	Detected	Non detected
**Individual 6**	F	64	NTNBC	64	Detected	Non detected
**Individual 7**	F	27	TNBC	27	Detected	**BRCA1 Del Ex 9-12**
**Individual 8**	F	61	NTNBC	60	Detected	Non detected
**Individual 9**	F	58	NTNBC	57	Detected	Non detected
**Individual 10**	F	46	TNBC	46	Detected	**PALB2 c.2411_2412del**

Table S2 Cancer frequency in non-analyzed relatives

	**Sex**	**Age**	**Cancer**	**Age of Dx**	**Reason for not analyzing**
**Family 1**					
**Paternal Uncle**	M	Unknown	Central Nervous System	Unknown	Death
**Paternal Cousin**	F	56	Ovarian	56	Death
**Family 2**					
**Maternal Aunt**	F	Unknown	Pancreas	Unknown	Death
**Maternal Aunt**	F	50	Colon	50	Death
**Brother**	M	44	Pancreas	44	Death
**Family 3**					
**Nephew**	M	13	Leukemia	13	Death
**Family 4**					
**Father**	M	61	Gastric	61	Death
**Mother**	F	59	No		Death
**Family 5**					

**Paternal Grandmother**	F	45	Thyroid	45	Death
**Maternal Aunt**	F	48	Pancreas	48	Death
**Maternal Aunt**	F	45	No		Death
			**Family 6**		
**Maternal Great Grandmother**	F	Unknown	Pulmonary	Unknown	Death
**Paternal Uncle**	M	Unknown	Lymphoma	Unknown	Death
			**Family 7**		
**Father**	M	52	No		Not interested
			**Family 10**		
**Mother**	F	53	Gastric	53	Death
**Maternal Grandmother**	F	56	Gastric	56	Death
**Paternal Grandmother**	F	52	Unknown Gastrointestinal	52	Death
			**Family 14**		
**Sibiling**	F	74	No		Not interested
**Sibiling**	M	78	No		Not interested
**Son**	M	46	No		Not interested
			**Unrelated individual 1**		
**Mother**	F	51	Central Nervous System	51	Death
**Father**	M	79	No		Death
**Sibiling**	F	56	Melanoma / Sarcoma / Breast	48 / 52/ 52	Pending
**Silbiling**	F	40	No		Pending
**Sibiling**	M	26	Melanoma	26	Death
**Sibiling**	F	7	Retinoblastoma	7	Death
**Son**	M	12	Dysplasic nevi	12	Mother choice
**Son**	M	15	No		Mother choice

Table S3 MINAS patients distribution

	Sex	Age	Cancer	Age of Dx	Variant 1	Variant 2
**Family 5**						
**Proband**	F	32	TNBC	30	CDKN2A (p16INK4a) c.146T>C (p.Ile49Thr)	BRCA1 c.5123C>A (p.Ala1708Glu)
**Sibling**	M	30	No		CDKN2A (p16INK4a) c.146T>C (p.Ile49Thr)	BRCA1 c.5123C>A (p.Ala1708Glu)
**Family 7**						
**Proband**	F	32	Melanoma	23/27	CDKN2A (p16INK4a) c.146T>C (p.Ile49Thr)	CDKN2A (p16INK4a) c.146T>C (p.Ile49Thr)
**Non related probands**						
**Individual 7**	F	27	TNBC	27	CDKN2A (p16INK4a) c.146T>C (p.Ile49Thr)	BRCA1 Del Ex 9-12
**Individual 10**	F	46	TNBC	46	CDKN2A (p16INK4a) c.146T>C (p.Ile49Thr)	PALB2 c.2411_2412del
